# Falta de Uso de Anticoagulantes em Pacientes com Fibrilação Atrial e Risco Aumentado de Eventos Tromboembólicos de Acordo com o Sexo: Insights de um Estudo Multicêntrico Brasileiro

**DOI:** 10.36660/abc.20240310

**Published:** 2024-09-17

**Authors:** Emiliano Medei, Renata Moll-Bernardes, Martha V. T. Pinheiro, Andréa S. Sousa, Barbara Abufaiad, Andre Feldman, Guilherme D’andrea Saba Arruda, Thiago Libano Csernik Monteiro, Fabio Augusto De Luca, Benhur Davi Henz, Denilson C. Albuquerque, Antonio Aurelio P. Fagundes, Marcia M. Noya-Rabelo, Angelina Silva Camiletti, Rose Mary Frajtag, Ronir R. Luiz, Olga F. Souza

**Affiliations:** 1 Instituto D’Or de Pesquisa e Ensino Rio de Janeiro RJ Brasil Instituto D’Or de Pesquisa e Ensino, Rio de Janeiro, RJ – Brasil; 2 Centro Nacional de Biologia Estrutural e Bioimagem Universidade Federal do Rio de Janeiro Rio de Janeiro RJ Brasil Centro Nacional de Biologia Estrutural e Bioimagem - Universidade Federal do Rio de Janeiro (UFRJ), Rio de Janeiro, RJ – Brasil; 3 Instituto de Biofísica Carlos Chagas Filho Universidade Federal do Rio de Janeiro Rio de Janeiro RJ Brasil Instituto de Biofísica Carlos Chagas Filho - Universidade Federal do Rio de Janeiro (UFRJ), Rio de Janeiro, RJ – Brasil; 4 Departamento de Cardiologia e Clínica Médica Rede D’Or Rio de Janeiro RJ Brasil Departamento de Cardiologia e Clínica Médica, Rede D’Or, Rio de Janeiro, RJ – Brasil; 5 Instituto Nacional de Infectologia Evandro Chagas Fundação Oswaldo Cruz Rio de Janeiro RJ Brasil Instituto Nacional de Infectologia Evandro Chagas, Fundação Oswaldo Cruz, Rio de Janeiro, RJ – Brasil; 6 Departamento de Cardiologia Universidade Estadual do Rio de Janeiro Rio de Janeiro RJ Brasil Departamento de Cardiologia, Universidade Estadual do Rio de Janeiro, Rio de Janeiro, RJ – Brasil; 7 Faculdade de Medicina e Saúde Pública da Bahia Salvador BA Brasil Faculdade de Medicina e Saúde Pública da Bahia, Salvador, BA – Brasil; 8 Instituto de Estudos em Saúde Pública Universidade Federal do Rio de Janeiro Rio de Janeiro RJ Brasil Instituto de Estudos em Saúde Pública (IESC) Universidade Federal do Rio de Janeiro (UFRJ), RRio de Janeiro, RJ – Brasil

**Keywords:** Fibrilação Atrial, Anticoagulantes, Tromboembolia

## Abstract

**Fundamento:**

A fibrilação atrial (FA) é a arritmia cardíaca mais prevalente e sua apresentação difere de acordo com a idade e o sexo. Estudos recentes revelaram diferenças na FA entre vários grupos demográficos, incluindo a população latino-americana.

**Objetivos:**

Melhor compreender as possíveis disparidades na prevalência da FA e nas estratégias de tratamento na população brasileira por meio de dados de um registro prospectivo multicêntrico de grande escala.

**Métodos:**

O registro de FA da Rede D’Or é um estudo observacional prospectivo multicêntrico que incluiu pacientes com idade ≥ 18 anos com FA atendidos no pronto-socorro de 32 hospitais terciários no Brasil. Os pacientes foram caracterizados de acordo com o sexo e outras características basais e classificados de acordo com o uso prévio de anticoagulantes. Foi analisada a falta de uso de anticoagulantes em pacientes com indicações prévias. A significância estatística foi estabelecida em 5%.

**Resultados:**

Os dados do estudo foram provenientes de um total de 1.955 pacientes inscritos. O sexo masculino foi mais prevalente e os homens eram mais jovens que as mulheres. Devido ao aumento da prevalência de episódios anteriores de FA e a um escore CHA2DS2-VASc mais elevado, mais mulheres tiveram indicação de terapia anticoagulante; no entanto, uma proporção significativa não estava recebendo esse tratamento. Dos 29 óbitos intra-hospitalares, 15 pacientes tinham indicação prévia para anticoagulação, mas apenas 3 estavam em uso de anticoagulantes.

**Conclusão:**

O presente estudo revelou diferenças relacionadas ao sexo na população brasileira de pacientes com FA que são consistentes com tendências em países de alta renda. A promoção de uma melhor implementação de terapias anticoagulantes e antitrombóticas para reduzir o risco de óbito e eventos tromboembólicos entre mulheres com FA no Brasil é crucial.

## Graphical abstract

**Figura Central:**
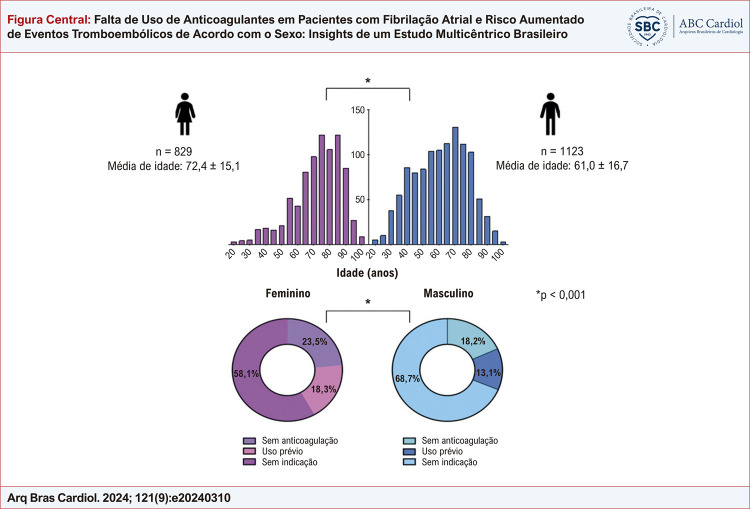
: Falta de Uso de Anticoagulantes em Pacientes com Fibrilação Atrial e Risco Aumentado de Eventos Tromboembólicos de Acordo com o Sexo: Insights de um Estudo Multicêntrico Brasileiro

Distribuição etária e uso de anticoagulantes de pacientes com fibrilação atrial de acordo com o sexo.

## Introdução

A fibrilação atrial (FA) é a arritmia cardíaca sustentada mais comum em todo o mundo e sua prevalência entre adultos no Brasil é de 1,8% a 2,5%, afetando aproximadamente 1,5 milhão de pessoas.^1-3^ O risco de FA ao longo da vida é de 25% e aumenta com a idade, os homens sendo mais frequentemente afetados. Outros fatores de risco conhecidos para o desenvolvimento e progressão da doença incluem hipertensão, diabetes, insuficiência cardíaca, miocardiopatia isquêmica, insuficiência renal crônica, doença pulmonar crônica, obesidade e apneia obstrutiva do sono.^4,5^

As complicações tromboembólicas, especialmente o acidente vascular cerebral (AVC), associadas à FA têm consequências graves, aumentando a morbimortalidade.^6^ Os pacientes com FA apresentam risco 5 vezes maior de AVC na ausência de tratamento anticoagulante. O risco de AVC durante a FA duplica a cada década de vida após a idade de 55 anos e a sua incidência ultrapassa 25% em pacientes com mais de 80 anos. A FA é responsável por quase um terço de todos casos de AVC e é a principal causa de AVC cardioembólico.^7,8^

As diretrizes atuais recomendam terapia antitrombótica para prevenção de AVC com base no risco individual.^4,9,10^ O escore CHA_2_DS_2_-VASc é o mais utilizado na prática clínica para prever o risco de AVC, com base nos riscos aumentados conferidos por insuficiência cardíaca, hipertensão, idade, diabetes, AVC prévio, doença aterosclerótica e sexo feminino. As mulheres com FA apresentam maior gravidade de AVC e ocorrência de incapacidade permanente do que os homens. Ainda assim, elas têm sido sub-representadas em ensaios clínicos randomizados sobre anticoagulantes orais não antagonistas da vitamina K (NOACs).^6,11^ Em relação aos pacientes do sexo masculino, as mulheres com FA têm menor probabilidade de serem submetidas à cardioversão elétrica e são encaminhadas mais tardiamente para ablação por cateter.^12^

A crescente prevalência e a complexidade da FA representam desafios clínicos significativos.^13^ As diferenças relacionadas ao sexo na FA podem ser atribuídas a variações no estilo de vida, perfis genéticos e hormonais, entre outros fatores. Até o momento, entretanto, essas diferenças não foram estudadas de forma abrangente em pacientes latino-americanos, incluindo a população brasileira. Assim, no presente estudo, objetivamos esclarecer as diferenças de idade e sexo nos aspectos epidemiológicos da FA e examinar a subutilização de anticoagulantes em pacientes com FA utilizando dados de um registro brasileiro prospectivo multicêntrico de grande escala.

## Métodos

### Desenho do estudo

Trata-se de um estudo observacional prospectivo multicêntrico denominado “registro de FA da Rede D’Or” de adultos consecutivos com idade ≥ 18 anos com FA sintomática, internados nas salas de emergência de 32 hospitais terciários em 6 estados brasileiros (Tabela S1).

### Participantes

Foram elegíveis para inclusão no registro pacientes com suspeita clínica, confirmada por eletrocardiograma de 12 derivações, de FA ou *flutter* atrial. Foram excluídos pacientes diagnosticados com taquiarritmias relacionadas a outras condições clínicas, como sepse e disfunção tireoidiana.

### Coleta e processamento de dados

Investigadores treinados coletaram dados demográficos, clínicos e laboratoriais dos prontuários médicos eletrônicos dos participantes e os inseriram em formulários eletrônicos de relato de caso usando a plataforma Research Electronic Data Capture (Vanderbilt University, Nashville, TN, EUA). Os dados clínicos incluíram história prévia de FA, comorbidades, apresentação clínica, fatores de risco cardioembólicos, história médica, uso prévio de anticoagulantes, procedimentos diagnósticos, complicações e tratamento durante a internação hospitalar. Os dados laboratoriais foram provenientes de exames realizados durante as internações dos participantes, de acordo com a prática clínica local. Os pacientes foram acompanhados prospectivamente até a alta hospitalar ou óbito intra-hospitalar.

Para o presente estudo, os pacientes foram caracterizados de acordo com o sexo e outras características basais e classificados de acordo com o uso prévio de anticoagulantes. Foi registrada e analisada a falta de uso de anticoagulante antes da admissão hospitalar em pacientes com indicação prévia para tal uso (história prévia de FA, escore CHA_2_DS_2_-VASc ≥ 3 para mulheres e ≥ 2 para homens).

### Análise estatística

As variáveis categóricas foram caracterizadas como proporções e suas frequências foram comparadas entre os grupos por meio do teste qui-quadrado ou de Fisher. A distribuição normal dos dados foi calculada pelo teste de Kolmogorov-Smirnov. Visto que as variáveis contínuas não apresentaram distribuição normal, foram descritas como medianas e intervalos interquartis e comparadas pelo teste de Mann-Whitney. A significância estatística foi estabelecida em 5%. Todas as análises foram realizadas utilizando o software SPSS (versão 24.0; IBM Corporation, Armonk, NY, EUA).

### Considerações éticas

O presente estudo segue os princípios da Declaração de Helsinque. O protocolo foi aprovado pelos conselhos de revisão institucional e pelos comitês de ética dos locais participantes (IRB#82452218.2.1001.5249). Todos os pacientes forneceram consentimento informado por escrito antes da inscrição. Todos os dados específicos dos pacientes foram desidentificados durante a análise para garantir a confidencialidade.

## Resultados

**Figura 1:**
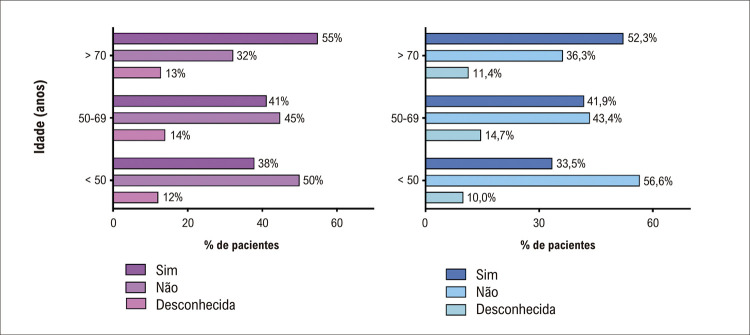
– História prévia de fibrilação atrial entre pacientes hospitalizados por fibrilação atrial de acordo com idade e sexo.

Entre 15 de junho de 2018 e 17 de fevereiro de 2023, foram incluídos no registro dados de um total de 1.955 pacientes dos 32 locais participantes. Destes pacientes, 707 apresentaram FA paroxística; 369 tinham FA permanente e a forma de FA não foi determinada em 879. A média de idade dos pacientes foi de 68,0 anos e 57,5% dos pacientes eram do sexo masculino. A idade variou de acordo com o sexo, com mediana de 61,0 anos para os homens e 72,4 anos para as mulheres (Figura Central). Mais mulheres do que homens tinham asma e hipertireoidismo. Mais homens do que mulheres tinham hipertensão e doença arterial coronariana. As frequências de insuficiência renal, dilatação e miocardiopatia isquêmica não diferiram entre os sexos (Tabela 1). História prévia de FA foi mais prevalente entre mulheres do que entre homens (49,6% versus 43,1%, p < 0,01), mas essa diferença não foi significativa quando a amostra foi estratificada por idade (Figura 1).

**Tabela 1:** – Características clínicas basais dos pacientes com fibrilação atrial de acordo com o sexo

Características	Total		Sexo				Teste χ^**2**^ Valor p
			Masculino		Feminino		
	n	(%)	n	(%)	n	(%)	
**Idade**							
< 50 anos	368	(18,9)	301	(26,8)	67	(8,1)	<0,001
De 50 a 69 anos	667	(34,2)	434	(38,6)	233	(28,1)	
> 70 anos	917	(47,0)	388	(34,6)	529	(63,8)	
**Hipertensão**							
Não	992	(55,6)	394	(51,8)	598	(58,5)	0,005
Sim	791	(44,4)	367	(48,2)	424	(41,5)	
**Doença arterial coronariana**							
Não	1504	(84,4)	844	(82,6)	660	(87,0)	0,012
Sim	277	(15,6)	178	(17,4)	99	(13,0)	
**Asma**							
Não	1714	(96,2)	996	(97,5)	718	(94,5)	0,001
Sim	68	(3,8)	26	(2,5)	42	(5,5)	
**Miocardiopatia dilatada/isquêmica**							
Não	1677	(94,2)	962	(94,1)	715	(94,2)	1.000
Sim	104	(5,8)	60	(5,9)	44	(5,8)	
**Insuficiência renal**							
Não	1717	(96,3)	982	(96,1)	735	(96,6)	0,614
Sim	66	(3,7)	40	(3,9)	26	(3,4)	
**Hipertireoidismo**							
Não	1751	(98,3)	1010	(98,8)	741	(97,5)	0,043
Sim	31	(1,7)	12	(1,2)	19	(2,5)	

### Mortalidade intra-hospitalar

Um total de 29 (1,48%) pacientes faleceu durante a internação hospitalar e apenas 1 (0,05%) faleceu na sala de emergência. A mortalidade esteve associada à idade, presença de comorbidades (p = 0,02), miocardiopatia dilatada ou isquêmica e insuficiência renal, mas não ao sexo, à hipertensão ou à doença arterial coronariana (Tabela 2). As causas de óbito foram cardiovasculares em 9 pacientes (incluindo 3 com insuficiência cardíaca, 1 com choque cardiogênico, 1 com AVC e 4 com outras causas cardiovasculares). A causa de óbito não foi relacionada a doenças cardiovasculares em 20 pacientes. É importante ressaltar que dos 29 óbitos, 15 (51,7%) pacientes tinham indicação prévia de anticoagulação, mas apenas 3 estavam em uso de anticoagulantes. A mortalidade no grupo com falta anticoagulação foi de 3,1%, comparada a 1,1% no grupo com anticoagulação prévia (p = 0,034). Não houve diferença entre os sexos nesse aspecto.

**Tabela 2:** – Mortalidade intra-hospitalar de pacientes com fibrilação atrial de acordo com características basais e comorbidades

Características	Total		Óbito				Valor p*
			Não		Sim		
	n	(%)	n	(%)	n	(%)	
**Idade**							
< 50 anos	321	100,0	320	(99,7)	1	(0,3)	0,002
De 50 a 69 anos	621	100,0	616	(99,2)	5	(0,8)	
> 70 anos	838	100,0	815	(97,3)	23	(2,7)	
**Sexo**							
Masculino	1032	100,0	1016	(98,4)	16	(1,6)	0,850
Feminino	748	100,0	735	(98,3)	13	(1,7)	
**Hipertensão**							
Não	906	100,0	890	(98,2)	16	(1,8)	0,852
Sim	731	100,0	719	(98,4)	12	(1,6)	
**Doença arterial coronariana**							
Não	1376	100,0	1355	(98,5)	21	(1,5)	0,191
Sim	260	100,0	253	(97,3)	7	(2,7)	
**Asma**							
Não	1573	100,0	1548	(98,4)	25	(1,6)	0,090
Sim	63	100,0	60	(95,2)	3	(4,8)	
**Miocardiopatia dilatada/isquêmica**							
Não	1540	100,0	1517	(98,5)	23	(1,5)	0,021
Sim	96	100,0	91	(94,8)	5	(5,2)	
**Insuficiência renal**							
Não	1576	100,0	1554	(98,6)	22	(1,4)	<0,001
Sim	61	100,0	55	(90,2)	6	(9,8)	
**Hipertireoidismo**							
Não	1609	100,0	1582	(98,3)	27	(1,7)	0,043
Sim	27	100,0	16	(59,3)	1	(3,7)	

*Teste de Fisher.

### Uso de anticoagulantes

A mediana do escore CHA_2_DS_2_-VASc foi 4 para mulheres e 2 para homens. Na população do estudo, 25,6% das mulheres apresentaram escore CHA_2_DS_2_-VASc ≥ 5; nos homens, essa proporção foi de 5,5% (Tabela S2). Considerando a história prévia de FA e o escore CHA_2_DS_2_-VASc, estimamos que 698 pacientes tinham indicação prévia de uso de anticoagulantes. Dentre esses pacientes, 299 (42,8%) relataram uso de anticoagulante na admissão hospitalar. Desses, 236 (78,9%) estavam em uso de NOAC; 15 (5,0%) estavam em uso de varfarina; 8 (2,6%) estavam em uso de heparina de baixo peso molecular e 40 (13,4%) não informaram. Não houve diferença significativa entre os sexos quanto ao tipo de anticoagulação (Tabela S3). Mais mulheres do que homens tinham indicação de uso de anticoagulantes (41,8% versus 31,3%) e não faziam uso (23,5% versus 18,2%; Figura Central).

Os 399 pacientes com falta de anticoagulação eram mais velhos e mais deles tinham hipertensão, doença arterial coronariana, asma, miocardiopatia dilatada ou isquêmica e insuficiência renal do que aqueles em uso prévio de anticoagulantes. A prevalência de hipertireoidismo não diferiu de acordo com o uso de anticoagulantes (Tabela 3). Entre todos os pacientes internados, 10 tiveram algum evento tromboembólico; 31 tiveram um evento hemorrágico e 1 paciente teve AVC hemorrágico.

**Tabela 3:** – Características clínicas de pacientes com fibrilação atrial segundo uso prévio de anticoagulantes

Características	Total		Anticoagulação prévia						Teste χ^**2**^ Valor p
			Sem indicação		Uso prévio		Falta de anticoagulação		
	n	(%)	n	(%)	n	(%)	n	(%)	
**Idade**									
< 50 anos	368	100,0	329	(89,4)	27,0	(7,3)	12,0	(3,3)	<0,001
De 50 a 69 anos	669	100,0	490	(73,2)	82,0	(12,3)	97,0	(14,5)	
> 70 anos	918	100,0	438	(47,7)	190,0	(20,7)	290,0	(31,6)	
**Hipertensão**									
Não	992	100,0	717	(72,3)	154	(15,5)	121	(12,2)	0,08
Sim	793	100,0	393	(49,6)	129	(16,3)	271	(34,2)	
**Doença arterial coronariana**									
Não	1506	100,0	1000	(66,4)	220	(14,6)	286	(19,0)	<0,001
Sim	277	100,0	109	(39,4)	62	(22,4)	106	(38,3)	
**Asma**									
Não	1716	100,0	1077	(62,8)	269	(15,7)	370	(21,6)	0,029
Sim	68	100,0	32	(47,1)	14	(20,6)	22	(32,4)	
**Miocardiopatia dilatada/isquêmica**									
Não	1679	100,0	1067	(63,5)	254	(15,1)	358	(21,3)	<0,001
Sim	104	100,0	42	(40,4)	28	(26,9)	34	(32,7)	
**Insuficiência renal**									
Não	1719	100,0	1082	(62,9)	267	(15,5)	370	(21,5)	0,04
Sim	66	100,0	28	(42,4)	16	(24,2)	22	(33,3)	
**Hipertireoidismo**									
Não	1753	100,0	1093	(62,4)	274	(15,6)	386	(22,0)	0,310
Sim	31	100,0	17	(54,8)	8	(25,8)	6	(19,4)	

Na alta hospitalar, um total de 1.196 pacientes estavam anticoagulados, 1.161 (97,1%) com NOAC e 35 (2,9%) com varfarina. Houve maior percentual de mulheres em uso de varfarina em relação aos homens (4,5% versus 1,8%; p = 0,007), sem diferença entre os sexos quanto ao uso de NOAC. Além disso, na alta hospitalar, 193 pacientes estavam recebendo medicamentos antiagregantes (Tabela S4).

## Discussão

Considerando que a FA é a arritmia cardíaca mais prevalente em todo o mundo, são necessários novos conhecimentos sobre as suas características epidemiológicas, incluindo disparidades de idade e sexo em âmbitos geográficos, raciais, culturais e econômicos específicos.^14^ Essa análise de dados de registro de 32 locais ao longo de 5 anos produziu resultados interessantes em relação a fatores demográficos, especificidades sexuais e subutilização de anticoagulantes na população brasileira com FA.

Nossa população tinha preponderância de homens, que eram mais jovens que as mulheres incluídas. Esse achado corrobora os de estudos anteriores de registros brasileiros, incluindo o estudo publicado em 2015 por Marcolino et al.^3^ e o recente estudo RECALL.^1^ Ademais, verificamos que a história prévia de FA foi mais prevalente entre as mulheres do que entre os homens. Consistentemente, Tanaka et al.^15^ relataram diferenças específicas entre sexos na recorrência de FA após a ablação, sugerindo que as mulheres são mais resistentes que os homens a esse tratamento.

A mortalidade intra-hospitalar foi associada à idade, mas não ao sexo, em nossa análise. A taxa de mortalidade intra-hospitalar no presente estudo (1,48%) foi superior àquela relatada para uma população europeia (0,6%),^16^ mas consistente com aquela observada em um estudo anterior de registro brasileiro.^3^ Observamos aumento da mortalidade no grupo de pacientes com falta de anticoagulação em comparação com aqueles que estavam em uso de anticoagulantes.

A avaliação adequada do risco tromboembólico e a implementação de estratégias de anticoagulação são vitais para prevenir AVC e outras complicações associadas em pacientes com FA.^17,18^ Estudos anteriores enfatizaram que as disparidades raciais, étnicas, sexuais e socioeconômicas podem afetar as estratégias de tratamento.^19^ Em nossa população, as mulheres eram mais propensas a necessitar de tratamento anticoagulante do que os homens. Portanto, uma taxa preocupante de 23,5% das mulheres de alto risco (ou seja, com história prévia de FA) não estavam recebendo terapia anticoagulante adequada. Essa questão, embora não seja exclusiva do Brasil, parece ser particularmente crítica na população brasileira, conforme apoiado por achados anteriores.^3,20^ Outras pesquisas, incluindo um estudo multicêntrico realizado no Japão^15^ e outro estudo realizado na Escócia,^21^ também revelaram discrepâncias baseadas no sexo na prescrição de anticoagulantes orais a pacientes com FA, possivelmente conferindo risco aumentado de AVC e hospitalização entre pacientes submedicados, embora associado a um menor risco de sangramento intracraniano. No entanto, outro registro de FA da China não descreveu diferença entre os sexos em relação ao uso de anticoagulantes orais.^22^

Os anticoagulantes orais diretos (DOACs) e a varfarina são os anticoagulantes mais utilizados em pacientes com FA.^23^ Nossos resultados também apresentaram perfil semelhante e os DOACs foram mais frequentemente indicados que a varfarina. Um grande estudo nos Estados Unidos, que incluiu mais de 430.000 pacientes, relatou que o uso de varfarina diminuiu de 52,4% para 17,7% de 2011 a 2020 entre adultos com FA.^24^ O mesmo estudo também observou que 1 em cada 3 pacientes de alto risco com FA não estava tomando nenhum anticoagulante. Em um grande estudo brasileiro (RECALL),^1^ os autores relataram que, de todos os pacientes que estavam recebendo anticoagulantes, 62,6% estavam em uso de antagonistas da vitamina K e 37,4% em uso de DOACs. Essas diferenças provavelmente refletem o nível socioeconômico que impede a utilização generalizada dos DOACs mais caros. A prática em hospitais privados brasileiros é semelhante àquela relatada em países de alta renda, contrastando com o uso predominante de varfarina em hospitais públicos, conforme demonstrado pelo estudo RECALL.

O presente estudo tem algumas limitações. Não foi possível estimar o escore HAS-BLED, o que nos impediu de determinar com precisão o número de pacientes que deveriam ter recebido anticoagulantes e não o fizeram. Como todos os pacientes inscritos eram de hospitais privados, a representatividade da população brasileira pode ser limitada, embora a Rede D’Or abarque um grande número de pacientes de diferentes níveis socioeconômicos e regiões do Brasil. Pesquisas futuras deverão focar na identificação de estratégias para otimizar o manejo de pacientes com FA e reduzir os riscos associados à doença.

## Conclusão

Os achados do presente estudo brasileiro estão alinhados com perfis semelhantes de pacientes com FA em países de alta renda, que revelam diferenças comparáveis baseadas no sexo. Embora a FA tenha sido mais prevalente entre os homens na nossa amostra, nossas análises mostraram que um número significativo de pacientes de alto risco, principalmente mulheres, não tinham uso prévio de anticoagulantes, resultando em um risco aumentado de complicações tromboembólicas associadas à FA. Portanto, devem ser feitos esforços intensivos para promover a adoção de terapias anticoagulantes e antitrombóticas adequadas, particularmente para mulheres.
