# Supplementary material for: Falta de Uso de Anticoagulantes em Pacientes com Fibrilação Atrial e Risco Aumentado de Eventos Tromboembólicos de Acordo com o Sexo: Insights de um Estudo Multicêntrico Brasileiro
Source: Arq Bras Cardiol. 2024 Sep 17;121(9):e20240310. [Article in Portuguese] doi: 10.36660/abc.20240310 (PMC11495564; doi:10.36660/abc.20240310)
Supplement: Translation

# Translation: Lack of Anticoagulant Use in Patients with Atrial Fibrillation and Increased Risk of Thromboembolic Events According to Sex: Insights from a Multicentric Brazilian Study

**DOI:** 10.36660/abc.20240310i

**Authors:** Emiliano Medei, Renata Moll-Bernardes, Martha V. T. Pinheiro, Andréa S. Sousa, Barbara Abufaiad, Andre Feldman, Guilherme D’andrea Saba Arruda, Thiago Libano Csernik Monteiro, Fabio Augusto De Luca, Benhur Davi Henz, Denilson C. Albuquerque, Antonio Aurelio P. Fagundes, Marcia M. Noya-Rabelo, Angelina Silva Camiletti, Rose Mary Frajtag, Ronir R. Luiz, Olga F. Souza

**Affiliations:** 1 Brasil Instituto D’Or de Pesquisa e Ensino, Rio de Janeiro, RJ – Brasil; 2 Brazil Centro Nacional de Biologia Estrutural e Bioimagem - Universidade Federal do Rio de Janeiro (UFRJ), Rio de Janeiro, RJ – Brazil; 3 Brazil Instituto de Biofísica Carlos Chagas Filho - Universidade Federal do Rio de Janeiro (UFRJ), Rio de Janeiro, RJ – Brazil; 4 Brazil Departamento de Cardiologia e Clínica Médica, Rede D’Or, Rio de janeiro, RJ – Brazil; 5 Brazil Instituto Nacional de Infectologia Evandro Chagas, Fundação Oswaldo Cruz, Rio de Janeiro, RJ – Brazil; 6 Brazil Departamento de Cardiologia, Universidade Estadual do Rio de Janeiro, Rio de Janeiro, RJ – Brazil; 7 Brazil Faculdade de Medicina e Saúde Pública da Bahia, Salvador, BA – Brazil; 8 Brasil Instituto de Estudos em Saúde Pública (IESC) Universidade Federal do Rio de Janeiro (UFRJ), Rio de Janeiro, RJ – Brasil

**Keywords:** Atrial Fibrillation, Anticoagulants, Thromboembolism

## Abstract

**Background:**

Atrial fibrillation (AF) is the most prevalent cardiac arrhythmia, and its presentation differs according to age and sex. Recent studies have revealed differences in AF among various demographic groups, including the Latin American population.

**Objectives:**

To better understand potential disparities in AF prevalence and treatment strategies in the Brazilian population through data from a large multicentric prospective registry.

**Methods:**

The Rede D’Or AF registry is a multicenter prospective observational study including patients aged ≥ 18 years with AF who were seen in the emergency department of 32 tertiary hospitals in Brazil. Patients were characterized according to sex and other baseline characteristics and were classified according to previous anticoagulant use. The lack of anticoagulant use in patients with previous indications was analyzed. Statistical significance was set at 5%.

**Results:**

The study data were from a total of 1955 patients enrolled. Male sex was more prevalent, and men were younger than the women. Due to an increased prevalence of previous AF episode and a higher CHA_2_DS_2_-VASc score, more women had indications for anticoagulant therapy; however, a significant proportion was not receiving this treatment. From 29 in-hospital deaths, 15 patients had previous indication for anticoagulation, but only 3 were using anticoagulants.

**Conclusion:**

This study revealed sex-related differences in the Brazilian population of patients with AF that are consistent with trends in high-income countries. The promotion of better implementation of anticoagulant and antithrombotic therapies to reduce the risk of death and thromboembolic events among women with AF in Brazil is crucial.

## Introduction

Atrial fibrillation (AF) is the most common sustained cardiac arrhythmia worldwide, and its prevalence among adults in Brazil is 1.8% to 2.5%, representing about 1.5 million people.^1-3^ The lifetime risk of AF is 25%, and it increases with age, with men being more commonly affected. Other known risk factors for the development and progression of the disease include hypertension, diabetes, heart failure, ischemic cardiomyopathy, chronic renal failure, chronic pulmonary disease, obesity, and obstructive sleep apnea.^4,5^

Thromboembolic complications, especially stroke, in association with AF have serious consequences, increasing morbidity and mortality.^6^ Patients with AF have a 5-fold increased risk of stroke in the absence of anticoagulant treatment. The risk of stroke during AF doubles with each decade of life after the age of 55 years, and its incidence exceeds 25% in patients over 80 years of age. AF accounts for almost one third of all strokes and is the leading cause of cardioembolic stroke.^7,8^

Current guidelines recommend antithrombotic therapy for stroke prevention based on individual risk.^4,9,10^ The CHA_2_DS_2_-VASc score is the most used in clinical practice to predict stroke risk, based on the increased risks conferred by heart failure, hypertension, age, diabetes, previous stroke, atherosclerotic disease, and female sex. Women with AF have greater stroke severity and permanent disability occurrence than do men. Still, they have been underrepresented in landmark randomized clinical trials of non–vitamin-K-antagonist oral anticoagulants (NOACs).^6,11^ Relative to male patients, women with AF are less likely to undergo electrical cardioversion and are referred later for catheter ablation.^12^

The increasing prevalence and the complexity of AF pose significant clinical challenges.^13^ Sex differences in AF could be attributed to variations in lifestyle, genetic and hormonal profiles, among other factors. To date, however, these differences have not been studied thoroughly in Latin American patients, including the Brazilian population. Thus, in the present study, we aimed to clarify the age and sex differences in the epidemiological aspects of AF and examine the underutilization of anticoagulants for patients with AF using data from a large-scale multicentric prospective Brazilian registry.

## Methods

### Study design

This is a multicenter prospective observational study called “Rede D’Or AF registry” of consecutive adults aged ≥ 18 years with symptomatic AF admitted to the emergency rooms of 32 tertiary hospitals in 6 Brazilian states (Table S1).

### Participants

Patients with clinical suspicion and 12-lead electrocardiographic confirmation of AF or atrial flutter were eligible for inclusion in the registry. Those diagnosed with tachyarrhythmias related to other clinical conditions, such as sepsis and thyroid dysfunction, were excluded.

### Data collection and management

Trained investigators collected demographic, clinical, and laboratory data from participants’ electronic medical records and entered them into electronic case report forms using the Research Electronic Data Capture platform (Vanderbilt University, Nashville, TN, USA). Clinical data included previous history of AF, comorbidities, clinical presentation, cardioembolic risk factors, medical history, previous use of anticoagulants, diagnostic procedures, complications, and treatment during hospitalization. Laboratory data were from tests performed throughout participants’ hospitalizations according to local clinical practice. The patients were followed prospectively until hospital discharge or in-hospital death.

For the present study, patients were characterized according to sex and other baseline characteristics and were classified according to previous anticoagulant use. The lack of anticoagulant use before hospital admission in patients with previous indications for such use (previous history of AF, CHA_2_DS_2_-VASc score ≥ 3 for women and ≥2 for men) was recorded and analyzed.

### Statistical analysis

Categorical variables were characterized as proportions, and their frequencies were compared between groups using the chi-squared or Fisher test. The normal distribution of the data was calculated by the Kolmogorov-Smirnov test. As continuous variables were not normally distributed, they were described as medians and interquartile ranges and were compared using the Mann-Whitney test. Statistical significance was set at 5%. All analyses were performed using SPSS software (version 24.0; IBM Corporation, Armonk, NY, USA).

### Ethical considerations

This study adheres to the principles of the Declaration of Helsinki. The protocol was approved by the institutional review boards and ethics committees at the participating sites (IRB#82452218.2.1001.5249). All patients provided written informed consent before enrollment. All patient-specific data were de-identified during analysis to ensure confidentiality.

## Results

Between June 15, 2018, and February 17, 2023, data from a total of 1955 patients from the 32 participating sites were included in the registry. Among these patients, 707 presented paroxysmal AF; 369 had permanent AF, and in 879 the AF form was not determined. The median patient age was 68.0 years, and 57.5% of the patients were male. The age varied according to sex, with median ages of 61.0 years for men and 72.4 years for women (Central Illustration). More women than men had asthma and hyperthyroidism. More men than women had hypertension and coronary artery disease. The frequencies of renal failure, dilatation, and ischemic myocardiopathy did not differ between sexes (Table 1). Previous histories of AF were more prevalent among women than among men (49.6% versus 43.1%, p < 0.01), but this difference was not significant when the sample was stratified by age (Figure 1).

**Table 1:** – Baseline clinical characteristics of patients with atrial fibrillation according to sex

Caracteristics	Total		Sex				χ^**2**^ Test p value
			Male		Female		
	n	(%)	n	(%)	n	(%)	
**Age**							
< 50 years	368	(18.9)	301	(26.8)	67	(8.1)	<0.001
50 to 69 years	667	(34.2)	434	(38.6)	233	(28.1)	
> 70 years	917	(47.0)	388	(34.6)	529	(63.8)	
**Hypertension**							
No	992	(55.6)	394	(51.8)	598	(58.5)	0.005
Yes	791	(44.4)	367	(48.2)	424	(41.5)	
**Coronary artery disease**							
No	1504	(84.4)	844	(82.6)	660	(87.0)	0.012
Yes	277	(15.6)	178	(17.4)	99	(13.0)	
**Asthma**							
No	1714	(96.2)	996	(97.5)	718	(94.5)	0.001
Yes	68	(3.8)	26	(2.5)	42	(5.5)	
**Dilated/ischemic cardiomyopathy**							
No	1677	(94.2)	962	(94.1)	715	(94.2)	1.000
Yes	104	(5.8)	60	(5.9)	44	(5.8)	
**Renal failure**							
No	1717	(96.3)	982	(96.1)	735	(96.6)	0.614
Yes	66	(3.7)	40	(3.9)	26	(3.4)	
**Hyperthyroidism**							
No	1751	(98.3)	1010	(98.8)	741	(97.5)	0.043
Yes	31	(1.7)	12	(1.2)	19	(2.5)	

**Figure 1:** – Previous history of atrial fibrillation among patients hospitalized for atrial fibrillation according to age and sex.

### In-hospital mortality

A total of 29 (1.48%) patients died during hospitalization, and only 1 (0.05%) died in the emergency department. Mortality was associated with age, the presence of comorbidities (p = 0.02), dilated or ischemic myocardiopathy, and renal failure, but not sex, hypertension, or coronary artery disease (Table 2). The causes of death were cardiovascular in 9 patients (including 3 with heart failure, 1 with cardiogenic shock, 1 with cerebrovascular accident, and 4 with other cardiovascular causes). The cause of death was non-related to cardiovascular disease in 20 patients. It is important to remark that from 29 deaths, 15 (51.7%) patients had previous indication for anticoagulation, but only 3 were using anticoagulants. The mortality in the group lacking anticoagulation was 3.1%, compared with 1.1% in the group with previous anticoagulation (p = 0.034). There was no difference between sexes in this regard.

**Table 2:** – In-hospital mortality of patients with atrial fibrillation according to baseline characteristics and comorbidities

Caracteristics	Total		Death				p value*
			No		Yes		
	n	(%)	n	(%)	n	(%)	
**Age**							
< 50 years	321	100.0	320	(99.7)	1	(0.3)	0.002
50 to 69 years	621	100.0	616	(99.2)	5	(0.8)	
> 70 years	838	100.0	815	(97.3)	23	(2.7)	
**Sex**							
Male	1032	100.0	1016	(98.4)	16	(1.6)	0.850
Female	748	100.0	735	(98.3)	13	(1.7)	
**Hypertension**							
No	906	100.0	890	(98.2)	16	(1.8)	0.852
Yes	731	100.0	719	(98.4)	12	(1.6)	
**Coronary artery disease**							
No	1376	100.0	1355	(98.5)	21	(1.5)	0.191
Yes	260	100.0	253	(97.3)	7	(2.7)	
**Asthma**							
No	1573	100.0	1548	(98.4)	25	(1.6)	0.090
Yes	63	100.0	60	(95.2)	3	(4.8)	
**Dilated/ischemic cardiomyopathy**							
No	1540	100.0	1517	(98.5)	23	(1.5)	0.021
Yes	96	100.0	91	(94.8)	5	(5.2)	
**Renal failure**							
No	1576	100.0	1554	(98.6)	22	(1.4)	<0.001
Yes	61	100.0	55	(90.2)	6	(9.8)	
**Hyperthyroidism**							
No	1609	100.0	1582	(98.3)	27	(1.7)	0.043
Yes	27	100.0	16	(59.3)	1	(3.7)	

*Fisher test.

### Anticoagulant use

The median CHA_2_DS_2_-VASc score was 4 for women and 2 for men. In the study population, 25.6% of the women showed a CHA_2_DS_2_-VASc score ≥ 5; in men, this proportion was 5.5% (Table S2). Considering the previous history of AF and the CHA_2_DS_2_-VASc score, we estimated that 698 patients had previous indications for anticoagulant use. Among these patients, 299 (42.8%) reported anticoagulant use on hospital admission. From these, 236 (78.9%) were on a NOAC; 15 (5.0%) were on warfarin; 8 (2.6%) were taking low-molecular-weight heparin, and 40 (13.4%) were non-informed. There was no significant difference between sex regarding the type of anticoagulation (Table S3). More women than men had indications for anticoagulant use (41.8% versus 31.3%) and were not using anticoagulants (23.5% versus 18.2%; Central Illustration).

The 399 patients lacking anticoagulation were older, and more of them had hypertension, coronary artery disease, asthma, dilated or ischemic myocardiopathy, and renal failure than did those previously taking anticoagulants. The prevalence of hyperthyroidism did not differ according to anticoagulant use (Table 3). Among all hospital-admitted patients, 10 had some thromboembolic event; 31 had a hemorrhagic event, and 1 patient had a hemorrhagic cerebrovascular accident.

**Table 3:** – Clinical characteristics of patients with atrial fibrillation according to previous anticoagulant use

Caracteristics	Total		Previous anticoagulation						χ^**2**^ Test p value
			No indication		Previous use		Lacking anticoagulation		
	n	(%)	n	(%)	n	(%)	n	(%)	
**No indication**									
< 50 years	368	100.0	329	(89.4)	27.0	(7.3)	12.0	(3.3)	<0.001
50 to 69 years	669	100.0	490	(73.2)	82.0	(12.3)	97.0	(14.5)	
> 70 years	918	100.0	438	(47.7)	190.0	(20.7)	290.0	(31.6)	
**Hypertension**									
No	992	100.0	717	(72.3)	154	(15.5)	121	(12.2)	0.08
Yes	793	100.0	393	(49.6)	129	(16.3)	271	(34.2)	
**Coronary artery disease**									
No	1506	100.0	1000	(66.4)	220	(14.6)	286	(19.0)	<0.001
Yes	277	100.0	109	(39.4)	62	(22.4)	106	(38.3)	
**Asthma**									
No	1716	100.0	1077	(62.8)	269	(15.7)	370	(21.6)	0.029
Yes	68	100.0	32	(47.1)	14	(20.6)	22	(32.4)	
**Dilated/ischemic cardiomyopathy**									
No	1679	100.0	1067	(63.5)	254	(15.1)	358	(21.3)	<0.001
Yes	104	100.0	42	(40.4)	28	(26.9)	34	(32.7)	
**Renal failure**									
No	1719	100.0	1082	(62.9)	267	(15.5)	370	(21.5)	0.04
Yes	66	100.0	28	(42.4)	16	(24.2)	22	(33.3)	
**Hyperthyroidism**									
No	1753	100.0	1093	(62.4)	274	(15.6)	386	(22.0)	0.310
Yes	31	100.0	17	(54.8)	8	(25.8)	6	(19.4)	

At hospital discharge, a total of 1196 patients were anticoagulated, 1161 (97.1%) with NOAC and 35 (2.9%) with warfarin. There was a higher percentage of women taking warfarin compared to men (4.5% versus 1.8%; p = 0.007), with no difference between sex regarding the use of NOAC. Also, at hospital discharge, 193 patients were receiving antiaggregant medications (Table S4).

## Discussion

As AF is the most prevalent cardiac arrhythmia worldwide, new insights into its epidemiological characteristics, including age and sex disparities in specific geographic, racial, cultural, and economic environments, are needed.^14^ This analysis of registry data from 32 sites over 5 years yielded interesting findings regarding demographic factors, sex specificities, and anticoagulant underutilization in the Brazilian population with AF.

This population had a preponderance of men, who were younger than the included women. This finding corroborates those from previous Brazilian registry studies, including the study published in 2015 by Marcolino et al.^3^ and the recent RECALL study.^1^ Additionally, we found that previous histories of AF were more prevalent among women than among men. Consistently, Tanaka et al.^15^ reported sex-specific differences in AF recurrence after ablation suggesting that women are more resistant than men to this treatment.

In-hospital mortality was associated with age, but not sex, in our analysis. The in-hospital mortality rate in this study (1.48%) was higher than the one reported for a European population (0.6%),^16^ but consistent with that observed in a previous Brazilian registry study.^3^ We observed increased mortality in the group of patients who were lacking anticoagulation compared to those who were taking anticoagulants.

Appropriate thromboembolic risk assessment and anticoagulation strategy implementation are vital to prevent stroke and other associated complications in patients with AF.^17,18^ Previous studies highlighted that racial, ethnic, sex, and socioeconomic disparities may affect treatment strategies.^19^ In our population, women were more likely than men to require anticoagulant treatment. Therefore, a concerning 23.5% of women at high risk (i.e., with previous histories of AF) were not receiving appropriate anticoagulant therapy. This issue, although not unique to Brazil, appears to be particularly critical in the Brazilian population, as supported by previous findings.^3,20^ Other works, including a multicentric study conducted in Japan^15^ and another study conducted in Scotland,^21^ have also revealed sex-based discrepancies in the prescription of oral anticoagulants to patients with AF, possibly conferring increased risk of stroke and hospitalization among undermedicated individuals, although associated with a lower risk of intracranial bleeding. Conversely, another registry of AF from China described no sex difference regarding the use of oral anticoagulants.^22^

Direct oral anticoagulants (DOACs) and warfarin are the most used anticoagulants in patients with AF.^23^ Our results also presented a similar profile, and DOACs were more frequently indicated than warfarin. A large study in USA, which included more than 430,000 patients, reported that warfarin use declined from 52.4% to 17.7% from 2011 to 2020 among adults with AF.^24^ The same study also remarked that 1 in 3 high-risk patients with AF was not on any anticoagulant. In a large Brazilian study (RECALL),^1^ the authors found that, from all patients who were receiving anticoagulants, 62.6% were taking vitamin K antagonists and 37.4% were taking DOACs. These differences probably reflect the socio-economic status that precludes the widespread use of the more expensive DOACs. The practice in private Brazilian hospitals is similar to the one reported in high-income countries, contrasting to the predominant use of warfarin in public hospitals, as shown in the RECALL study.

The study has some limitations. It was not possible to estimate the HAS-BLED score, which prevented us from precisely determining the number of patients who should have received anticoagulants but did not. Since all patients enrolled were from private hospitals, the representativeness of the Brazilian population could be limited, even though Rede D’Or involves a large number of patients from different socioeconomic backgrounds and regions of Brazil. Future research should focus on the identification of strategies to optimize the management of patients with AF and reduce risks associated with the disease.

## Conclusion

The findings from the present Brazilian study are in line with similar profiles of patients with AF in high-income countries, which reveal comparable sex-based differences. Although AF was more prevalent among men in our sample, our analyses showed that a significant number of high-risk patients, particularly women, had no previous anticoagulant use, resulting in an increased risk of thromboembolic complications associated with AF. Thus, intensive efforts must be made to promote the adoption of appropriate anticoagulant and antithrombotic therapies, particularly for women.
